# Fabrication of Second Skin from Keratin and Melanin

**DOI:** 10.3390/polym12112568

**Published:** 2020-11-02

**Authors:** Chen Nowogrodski, Ido Simon, Shlomo Magdassi, Oded Shoseyov

**Affiliations:** 1 Plant Molecular Biology and Nano Biotechnology, Faculty of Agriculture, Food and Environment, The Hebrew University of Jerusalem, Rehovot 7610001, Israel; ido.simon@mail.huji.ac.il (I.S.); Oded.Shoseyov@mail.huji.ac.il (O.S.); 2 Casali Center of Applied Chemistry, Institute of Chemistry, The Hebrew University of Jerusalem, Jerusalem 91905, Israel; magdassi@mail.huji.ac.il

**Keywords:** wearable skin, keratin, melanin, films, cross-linking, bio-based, second skin

## Abstract

Second skin is a topically applied, skin-conforming material that mimics human skin properties and bears potential cosmetic and e-skin applications. To successfully integrate with natural skin, characteristics such as color and skin features must be matched. In this work, we prepared bio-based skin-like films from cross-linked keratin/melanin films (KMFs), using a simple fabrication method and non-toxic materials. The films retained their stability in aqueous solutions, showed skin-like mechanical properties, and were homogenous and handleable, with non-granular surfaces and a notable cross-linked structure as determined by attenuated total reflection (ATR). In addition, the combination of keratin and melanin allowed for adjustable tones similar to those of natural human skin. Furthermore, KMFs showed light transmittance and UV-blocking (up to 99%) as a function of melanin content. Finally, keratin/melanin ink (KMI) was used to inkjet-print high-resolution images with natural skin pigmented features. The KMFs and KMI may offer advanced solutions as e-skin or cosmetics platforms.

## 1. Introduction

The skin is a versatile organ with multiple physiological functions that is responsive to a fluctuating environment and largely affected by aging, environmental conditions, and diseases. Second skin is a topically applied, wearable, skin-conforming material that has been developed to address the immense interest in the electronic skin (e-skin) and the cosmetic industries [1,2,3,4], mimicking human skin and its complex properties, e.g., elasticity, tribology, and tactile sensing. Ideal “second skin” films should accurately replicate the stratum corneum (SC) layer, which is the top-most, visible layer of the skin which significantly influences skin appearance and dictates skin characteristics such as texture and color. The SC layer is responsible for the smooth, shiny, and soft look of healthy skin. Polydimethylsiloxane (PDMS) is a key second skin material because of its elastic properties, durability, and non-toxicity. However, its critical downsides include costliness and non-biodegradability [5,6]. Various materials, particularly biopolymers such as collagen, elastin, and keratin, have been suggested as potential alternatives for the development of bio-inspired artificial skin [3,7]. Although the incorporation of color and skin characteristics into wearable skin to provide its desired appearance is obvious, focus on aesthetic properties has fallen short.

Skin color is determined by the individual typology angle (ITA), which consists of six skin pigmentation groups: ITA value ˃55° (very light), 41°–55° (light), 28°–41° (intermediate), 10°–28° (tan), −30°–10° (brown), and <−30° (dark). The ITA is determined by the International Commission on Illumination as CIE L*a*b* values, which is a standardized method of modeling color appearance that better replicates human color perception—L*, lightness–darkness (0–100); a*, red (+)–green (−); and b*, yellow (+)–blue (−) [8,9,10]. In humans, skin color is dictated by melanin content, and apart from the determined human eye, hair, and pigmentation, melanin expression leads to the formation of beauty marks, freckles, and birthmarks and therefore has a crucial role in aesthetic appearance [3,10]. Melanin is a surface pigment that holds a broad spectral absorbance, enabling it to serve as a photoprotective agent against UV radiation in humans, and it has been suggested to provide structural strengthening by cross-linking proteins and protecting them from degradation [11,12]. Today, melanin is used in photoprotective creams, biomimetic structural color materials, and e-inks [13,14,15]. 

Keratins can be found in the SC, in addition to dead keratinocytes, melanin pigments, and lipids [16,17,18]. Keratins are roughly divided into soft and hard keratins; hard keratins that are present in hair, wool, and feathers are classified as non-soluble fibrous proteins that bear large numbers of disulfide bonds, which contribute to their structural stiffness. The biological activity, biocompatibility, and biodegradability of keratins have led to their integration in biomedical applications such as wound healing, tissue engineering, skin scaffolds, drug delivery, and also in the fabrication of hydrogels, fibers, composites, and inks [19,20,21,22,23,24,25]. Keratin films have been well studied, mainly in the fields of regenerative medicine and food packaging [26,27,28,29]. 

Previous work with synthetic melanin and similar compounds gives tunable color characteristics holding potential applications in the field of cosmetics [30,31]. Several studies incorporate keratin and melanin. However, those studies are yet to focus on aesthetic features for wearable skin. For instance, an earlier work of keratin sponges with polydopamine (PDA) nanoparticles reported antioxidant properties [32], while a recent work examined PDA-functionalized keratin films as a photoactive coating material [33]. A different work demonstrated the feasibility of applying a two-step, inkjet bioprinting-based strategy to fabricate a 3D-pigmented human skin construct with uniform skin pigmentation [3]. Additionally, second skin with realistic human features, as well as high-resolution features, has preferable rates of adoption by end users [34]. Since second skin can serve as a cosmetic cover, there is an unmet need for second skin to suit a variety of human pigmentation ranges, both for purely aesthetic purposes and for end-user satisfaction and inclusivity. Therefore, we designed, developed, and characterized novel SC-conforming, cross-linked keratin/melanin films (KMFs) to serve as a second skin with tunable tones and skin characteristics. Based on the composition of the KMFs, a keratin/melanin ink (KMI) was also developed to enable precise, high-resolution inkjet printing of second skin textures and features that are unachievable with traditional film casting and can help drive end-user satisfaction and adoption.

## 2. Materials and Methods 

### 2.1. Materials

Raw cashmere wool was purchased from a local supplier (Ness-Ziona, Israel). Urea, thiourea, 2-amino-2-hydroxymethyl-propane-1,3-diol (Tris), 2-mercaptoethanol, glycerol, Ellman’s reagent (5,50-dithiobis-(2-nitrobenzoic acid)), Sigmacote^®^, and synthetic melanin were purchased from Sigma (Rehovot, Israel). Byk 348 was donated by Byk Chemie (Maiko Engineering, Israel). Dialysis membranes (MWCO 12–14 kDa) were purchased from Medicell (London, England). All other chemicals used were of analytical grade. All solutions were formulated using deionized water (DW).

### 2.2. KMFs Preparation

#### 2.2.1. Keratin Extraction Procedure

Keratin was extracted according to the Shindai method [26,35] with some modifications. Raw, white cashmere wool (30 g) was rinsed three times, for 10 min each, in DW (5 L, room temperature (RT)), blotted and dried overnight in a 37 °C incubator. Then, lipids were extracted via a 25 h incubation in 100% acetone to remove unbound surface lipids (5 L, RT). Then, de-lipidized wool was washed three times, for 45 min each, with DW (5L, RT), and then blotted and dried overnight in a 37 °C incubator. Then, wool fibers were cut into short fibers about 3 mm in length, which were mixed with an aqueous extraction medium (400 mL) containing 25 mM Tris pH 8.5, 2.6 M thiourea, 5 M urea, and 5% 2-mercaptoethanol (50 °C for 72 h) to solubilize the fibers. Then, the mixture was centrifuged at 4500 *g* for 30 min, followed by supernatant filtration through a stainless-steel sieve (#200). The filtrate (‘‘Shindai extract’’) was exhaustively dialyzed (12–14 kDa cut-off) against DW (1:50) for 96 h at RT, with the dialysis fluid changed every 24 h. The dialysis was repeated until no 2-mercaptoethanol (via Ellman’s reagent) was detected in the dialysate. Then, the Shindai extract solution was centrifuged twice at 10,000 *g* for 30 min, resulting in an approximate concentration of 30 mg/mL of keratin, which was stored at −20 °C. Keratin samples were validated by Laemmli SDS-PAGE and the Bradford colorimetric method.

#### 2.2.2. Preparation of Water-Soluble Melanin

Synthetic melanin powder was dissolved in DW at 2 mg/mL, followed by a pH adjustment (Hanna Instruments, Woonsocket, RI, USA) to 11 (using NaOH 1 M buffer) to obtain water-soluble melanin. Then, samples were sonicated for 10 min [15,36] and filtered through a 0.2 µm Teflon filter immediately before use.

#### 2.2.3. KMFs Preparation

Keratin solution was thawed and adjusted to pH 9.5 (using NaOH 1 M buffer), incubated for 72 h at 60 °C, and filtered through a 0.2 µm Teflon filter immediately before use. Then, keratin and melanin were blended in the following ratios, respectively: 40:1 (KMF40), 100:1 (KMF100), 250:1 (KMF250), 500:1 (KMF500), and 1:0 (KMF0). Then, 2% (*w/v*) glycerol was added. The blends were gently mixed and degassed by centrifugation at 4000 *g* for 15 min, yielding a clear solution. Then, the blends were cast on Sigmacote-treated glass slides and left to dry overnight at RT, resulting in a gradient of transparent to deep brown KMFs. Thereafter, films were incubated at 110 °C for 3 h [27,28]. All films were allowed to cool to RT and stored in a desiccator.

### 2.3. Characterization of KMFs

#### 2.3.1. Attenuated Total Reflection (ATR)

Attenuated total reflection (ATR) spectra (400 to 4000 cm^−1^) of all KMFs were collected using an FT-IR Nicolet 6700 (Thermo scientific™, Waltham, MA, USA) equipped with an ATR accessory. The specimens were measured as films. Reference samples were keratin and melanin films without glycerol. All spectra were measured at a resolution of 8 cm^−1^, with 100 scans.

#### 2.3.2. High-Resolution Scanning Electron Microscopy (SEM)

SEM (Sirion, Thermo Scientific™, Waltham, MA, USA) was used to image cross-sectional and topographical morphologies of the films at 3 kV accelerating voltage (specimens were 10 nm gold-palladium-coated). Films were immersed in liquid nitrogen and fractured for cross-section views. 

#### 2.3.3. Water Absorption and Mass Loss

The water absorption capacities of the KMFs were assessed with three 10 mm × 10 mm square samples, which were immersed in DW at RT and gently blotted, weighed, and submerged again every 24 h, for one week. The following equation was used to calculate the gravimetric water absorption: $\frac{\mathrm{Ww}-\mathrm{Wdi}}{\mathrm{Wdi}}\times 100=\%\;\mathrm{Absorption}$, where Ww and Wdi respectively represent the wet weight and initial dry weight of the samples. Mass loss was measured both gravimetrically and spectroscopically. Specimens (10 mm × 5 mm) were measured after one week of overnight incubation at 40 °C (for water evaporation) and weighed. The following equation was used to calculate gravimetrically measured mass loss: $\frac{\mathrm{Wd}}{\mathrm{Wdi}}\times 100=\%\;\mathrm{Loss}$, where Wd is the final dry weight of the samples. Keratin traces in the aqueous phase were determined using the Bradford colorimetric method, while melanin traces were determined by UV-VIS spectrophotometry at 290 nm. 

#### 2.3.4. Mechanical Properties

The mechanical properties of specimens were determined by tensile tests with an Instron testing machine (Model 2519-107, Blue Hill 2.3 Software, Instron, Norwood, MA, USA), with a 100 N static load cell using six replicates. For this, the films were cut in rectangles of 5 mm × 15 mm and fixed on the grips of the device with a gap of 10 mm. The thickness of the sample, determined with an uncertainty of ±5 µm, was in the range of 50–70 µm. The films were tested at a speed of 10 mm per minute. 

### 2.4. Optical Measurements

#### 2.4.1. Light Transmittance 

A UV-VIS spectrophotometer (Thermo-Evolution 300, Thermo Scientific™, Waltham, MA, USA) was used to evaluate light transmission between 300 and 800 nm, at a resolution of 1 nm, with three repetitions. Specimens were cut to 20 mm × 10 mm strips and attached to the cell wall. UV-blockage was calculated by the approximate total area under the averaged curve from 300–400 nm (Riemann sum), which was divided by the total possible transmission over that range (100% × 100 nm). Then, the percentage transmission in the near UV range was converted to UV percentage of rejection. 

#### 2.4.2. Colorimetric Measurements 

KMFs and printed KMI were colorimetrically measured using an X-Rite i1 Pro instrument (X-Rite, Grand Rapids, MI, USA). Chromaticity was plotted by the MATLAB (The Mathworks Inc., Matlab, R2017b (chromaticity coordinate calculator), 2020, Natick, MA, USA), and ITA° values were calculated by the following equation: ${[tan}^{-1}(\frac{L*-50}{b*})]\times \frac{180}{\pi }$, where L* and b* values are from the 1931 CIE L*a*b* values.

### 2.5. Inkjet Printing of KMI

#### 2.5.1. KMI Preparation

Keratin solution was thawed and adjusted to pH 9.5 (using NaOH 1 M buffer), incubated for 72 h at 60 °C, and filtered through a 0.2 µm Teflon filter immediately before use. Then, keratin was blended with 15% (*w/v*) glycerol and 0.1% Byk 348, in order to form keratin ink. Melanin ink was composed of 2 mg/mL melanin, 15% (*w/v*) glycerol, 0.1% Byk 348, and filtered through a 0.2 µm Teflon filter immediately before use.

#### 2.5.2. Inkjet Printing

Melanin and keratin inks were loaded into two different cartridges: an Epson L310 home printer (Epson, Suwa-shi, Japan) and an Epson Micro Piezo™ print head (Epson, Suwa-shi, Japan) with a printing resolution of 5760 × 1440 dots per inch (DPI). The selected image was converted into two colors that corresponded to the fixed cyan, magenta, yellow, and key (black) (CMYK) colors of the cartridges. Then, the inks were printed on Epson paper, resulting in KMI-printed images.

## 3. Results

### 3.1. Characterization of the KMFs

#### 3.1.1. Fabrication of KMFs

The extraction of keratin proteins from cashmere wool yielded approximately 25 mg/mL, which presented on a typical SDS-PAGE as bands of keratins (soft and hard) in the range of 30–60 kDa (Figure S1). During KMF fabrication, keratin and melanin aggregation was avoided by working under alkaline pH conditions, which prevented the reformation of keratin disulfide bonds and melanin precipitation [36], and maintained the clarity and homogeneity of the keratin/melanin solutions. The addition of glycerol to the fabrication process resulted in homogenous, non-brittle, and handleable films which were of shades ranging from transparent to dark brown. As the melanin concentration of KMFs increased, the film transparency decreased. The average film KMF thickness was 40 µm, which is similar to that of the native SC layer. 

#### 3.1.2. Ultrastructure of KFMs

**Figure 1:**
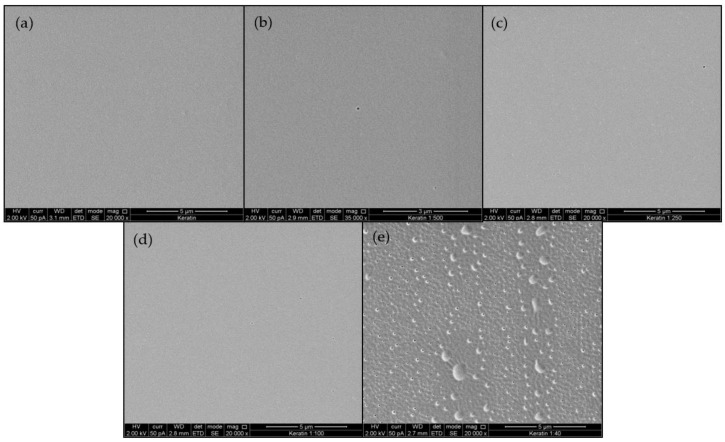
SEM imaging of the keratin/melanin films (KMFs) sequentially showing a smooth to perforated film surface as the ratio between keratin and melanin increases (**a**–**e**): KMF0, KMF500, KMF250, KMF100, and KMF40.

Figure 1 presents a typical SEM image of KMFs. Film roughness ranged from smooth, plain, and homogeneous surfaces in KMF0 samples (Figure 1a), to porous and perforative microstructures in the KMF40 samples (Figure 1e). KMF40 samples exhibited a rough and uneven surface. Further analysis of film cross-sections after fracture demonstrated that the internal structure throughout the entire film volume was not compromised by the presence of melanin (Figure S2). 

#### 3.1.3. Structural Analysis of KFMs

**Figure 2:**
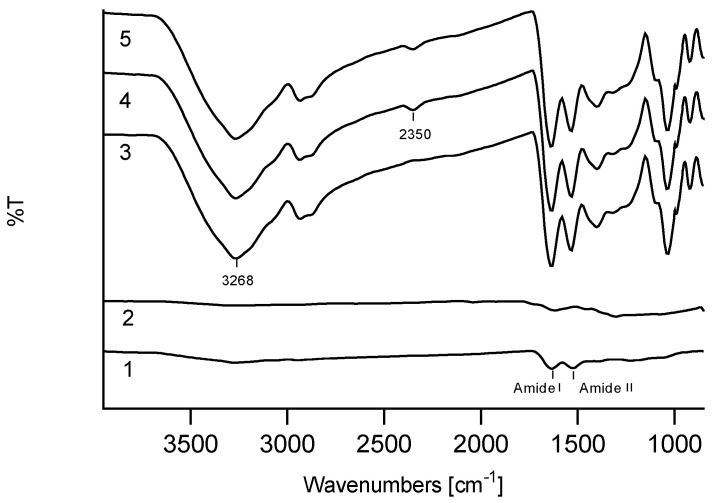
Attenuated total reflection (ATR) spectra of: (**1**) keratin, (**2**) melanin, (**3**) KMF0, (**4**) KMF250, (**5**) KMF40.

ATR analysis of the keratin/melanin interactions in the presence of glycerol showed characteristic keratin peaks, mainly assigned to amide I (1580–1680 cm^−1^) and amide II bands (1480–1580 cm^−1^), ascribed to C=O stretching vibrations, and to N–H bending and C–N stretching vibrations, respectively (Figure 2). In addition, cysteine-S-sulfonated residues at peaks 1097 cm^−1^ and 1034 cm^−1^ were due to asymmetric and symmetric S=O stretching vibrations, respectively (Figure S3a) [33,37]. The ATR peaks of all KMFs were significantly increased after the addition of glycerol. A double peak at 2800 cm^−1^ was assigned to the C–C–H and C–H groups of glycerol, and peaks sharpening at 3268 cm^−1^ were assigned to the stretching vibration of N–H in amino acids. 

The melanin spectrum showed a broad peak associated with OH groups above 3000 cm^−1^ and a shoulder peak at 1600 cm^−1^, which features C–N stretching of pyrrole or indole aromatic rings (Figure S3b). The KMF500-40 spectra showed a notable peak at 2350 cm^−1^, which was characteristic of the C=N bond [38], which could have originated from new bonds between keratin and melanin.

#### 3.1.4. Mechanical Properties and Water Stability 

**Figure 3:**
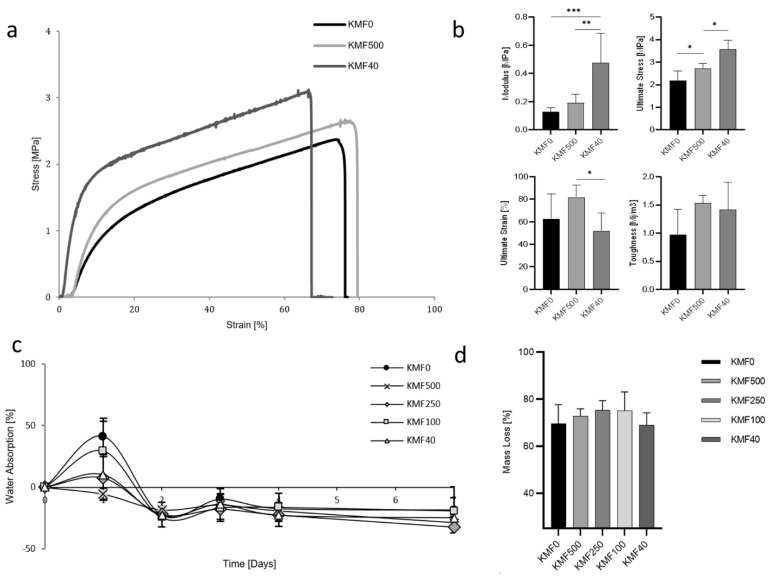
Mechanical properties and water stability of KMFs: (**a**) stress–strain curves of KMF0, KMF500, and KMF40. (**b**) Mechanical parameters: Young’s modulus, ultimate stress, ultimate strain, and toughness of KMF0, KMF500, and KMF40 and their statistically significant differences (* *p* < 0.05, ** *p* < 0.01, *** *p* < 0.001). (**c**) Water absorption over one week of KMFs submersion in water. (**d**) Mass loss of dried KMFs after immersion in water for one week.

The mechanical properties of three representative KMFs were examined by stress–strain curves and values of Young’s modulus, ultimate stress, ultimate strain, and toughness were analyzed (Figure 3a,b). The stress–strain curve profiles increased after reaching the yield point and until rupture. Furthermore, the elastic region and ultimate stress were increased as a function of the melanin addition, along with elongation at break reduction. The modulus varied from 0.13 to 0.48 MPa and was significantly influenced from melanin addition, as expected. The ultimate strain and the toughness (the area under the strain–stress curves) slightly dropped for KMF40.

Film durability was expected to significantly decline in an aqueous environment due to the water-soluble starting materials, lack of cross-linkers, and high keratin/glycerol ratio (1:1 *w/v*). Maximal water absorption varied between 0% and 40%, which was followed by final negative water absorption (Figure 3c). KMF0 and KMF40, which possessed the lowest and highest melanin content, respectively, exhibited the highest stability in water. 

A mass loss of 70% was measured, with no statistically significant differences between the tested KMFs (Figure 3d). The analyzed aqueous phase, which appeared clear and transparent after film removal, showed on average 5% keratin and 4% melanin (Table 1). 

### 3.2. Optical Properties of KMFs 

**Figure 4:**
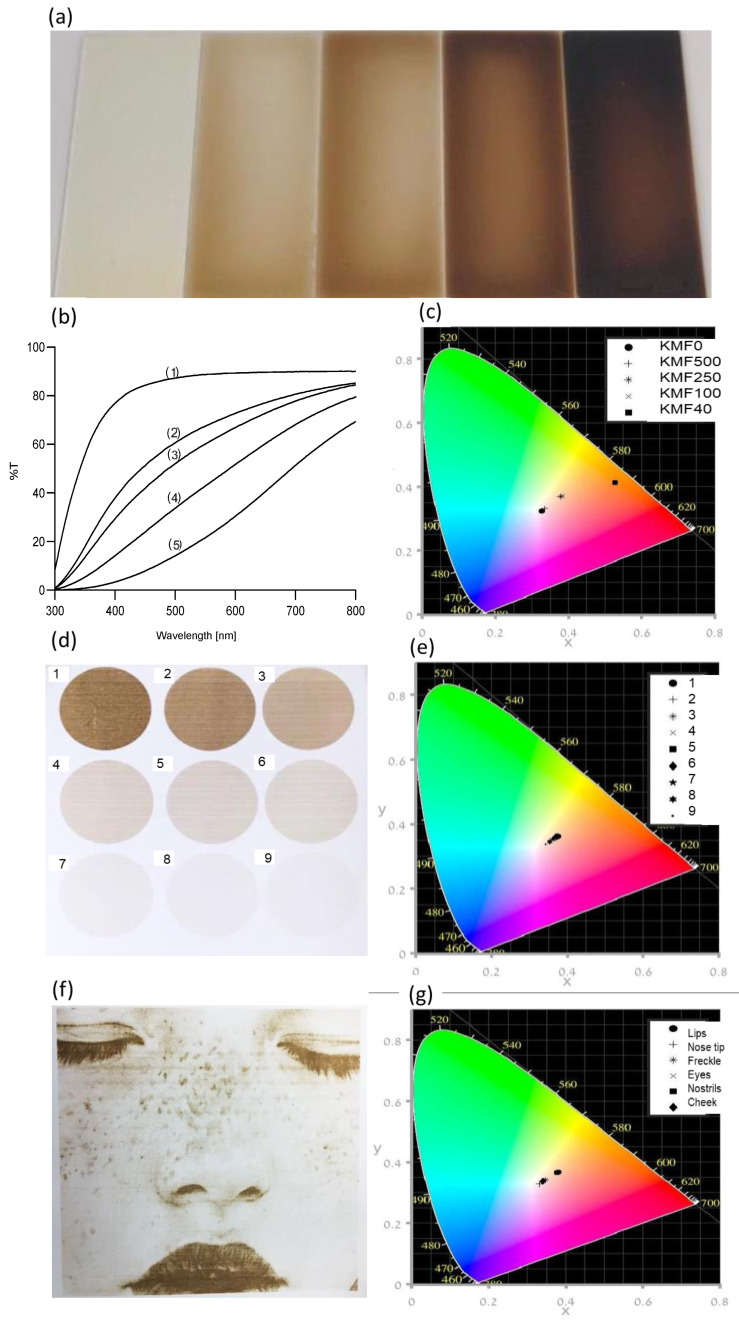
The optical properties of KMFs and keratin/melanin ink (KMI). (**a**) Keratin/melanin films with an increasing melanin concentration: KMF0, KMF500, KMF250, KMF100, and KMF40 (left to right). (**b**) UV-VIS transmission spectra of: (1) KMF0, (2) KMF500, (3) KMF250, (4) KMF100, and (5) KMF40. (**c**) The color changes for KMFs as a function of melanin content, as presented in the CIE 1931 color space. (**d**) Inkjet-printed skin color gradient (1–9 colors) from KMI. (**e**) The color changes of inkjet-printed skin color gradient, as presented in the CIE 1931 color space. (**f**) Inkjet-printed image of a face holding skin characteristics with KMI on Epson paper. (**g**) The color changes in different areas in the inkjet-printed face, as presented in the CIE 1931 color space.

As previously described, the KMFs showed a color gradient from transparent to dark-brown (Figure 4a). Light transmission and UV-blocking capacities of KMFs are presented in Figure 4b and Table 2, respectively. Light transmission of KMFs showed a strong and clear melanin dependence. More specifically, transmission percentages were highly affected by melanin concentration. KMF0 was nearly a completely transparent non-pigmented film, with a transmittance of 90%. As melanin concentrations increased and the film became darker, light transmission declined by up to 68%. Although KMF0 was notably transparent, the percentage of UV blockage was 40%; in KMF500, UV blockage was surprisingly almost double that of KMF0 and almost fully blocked in KMF40. Melanin addition caused color shifts toward the orange region of the spectrum of visible light that is described in Figure 4c as well as an almost linear trend from KMF0 to KMF40 of white (no color) to orange. When analyzing the CIE L*a*b values that also consider brightness, the addition of melanin led to a decrease in the ‘L’ value toward black, with increases in ‘a’ toward the red color and ‘b’ toward yellow. The ITA° values showed that KMF250 and KMF100 complied with light skin pigment and KMF40 with dark skin pigment definitions. Both CIE L*a*b and ITA° are described in Table 2.

### 3.3. Inkjet Printing of KMI Solutions

Keratin ink was clear and well dispersed with maximal particle sizes of 1 µm—in compliance with inkjet printing requirements. KMI was successfully printed with a color gradient on Epson paper as nine circles with slightly different melanin contents, which caused the pigmentation (numbered 1–9, where 1 had the highest melanin content), as shown in Figure 4d. The chromaticity in Figure 4e showed a similar trend to KMFs but holding lower ITA° values (˃55°). In addition, Figure 4f exhibits an image of a face bearing typical skin characteristics [39], such as freckles, which were printed to emphasize the potential use of the ink as a high-resolution additive in combination with KMFs for an aesthetically pleasing and unique second skin. The estimated layer thickness of the printed KMI is 100 nm (Figure S4). It was also observed that the KMI was firmly attached to the Epson paper and could not be detached as KMFs. The selected areas for CIE L*a*b* analysis (lips, eyes, etc.) also showed skin color that matched ITA° values.

## 4. Discussion

In recent years, an increasing number of studies have shown that keratin bears high potential for use in regenerative medicine, particularly in the skincare arena [7,19,40]. Keratin films have been previously fabricated by solvent casting and other methods, and they have also been blended with various materials such as hyaluronic acid, silk, and polyvinyl alcohol (PVA)—mainly for tissue engineering but yet for wearable skin [28,41,42]. Given the layered structure of the epidermis, which is flat and thin, the keratin film is a potential candidate material for the preparation of second skin [43]. During the course of the research, homogenous, flexible, and robust keratin/melanin films were fabricated and evaluated for the first time with emphasis on pigmentation through melanin content. The different keratin/melanin ratios yielded an adjustable color gradient that complied with ITA° values, which are natural human skin tones, and also provided ample UV blockage, skin-like mechanical properties, and sufficient water stability. The KMFs are water based and are composed of non-toxic materials. The cytotoxicity of keratin films was evaluated in several studies with no reported evidence of cytotoxicity [27]. Keratin films that were coated with PDA and evaluated for cyto-compatibility were also found to be bio-compatible [33]. KMFs were obtained after the removal of toxic substances from the Shindai extract via aqueous dialysis, the subsequent addition of melanin and glycerol, solvent casting, and drying. The choice of working pH strongly influenced the obtained film homogeneity, with an alkaline pH leading to the high solubility of keratin and melanin, and a neutral pH resulting in an opaque keratin solution and melanin aggregation. The neutral pH allowed for the oxidation of cysteine residues, resulting in the reformation of keratin disulfide bonds which yielded keratin particles. Melanin solubility is a function of the state of its carboxylic acid group, which is ionized at a high pH [36].

In nature, the keratin microstructure is well organized and underlies its final function, e.g., hair and nails [42]. According to our hypothesis, the slow and harsh water evaporation events that occur during solvent casting, fabrication, and curing allowed for the formation of disulfide, amine, amide, hydrophobic, and hydrogen bonds. The hydrolyzed keratin showed a known tightly packed, spherical nanoparticle surface [28]. On the other hand, the addition of melanin induced a moderate morphological imperfection in the form of nanometric pores. KMF40 exhibited a significantly different morphology compared to other KMFs, which may have been a result of the difficulty of water evaporation due to melanin’s hydrophobicity. An analysis of KMF cross-sections suggests that the entirety of the internal structure was not affected by the addition of melanin, and that the nanometric pores formed were only a film surface event. 

Interactions between keratin and the indole amino groups of PDA, as well as the successful polymerization of dopamine on the keratin surface have been previously reported [32,33]. The ATR spectra of KMF250 and KMF40 at 2350 cm^−1^ contained a notable peak of C=N which could have been the result of a reaction between melanin’s carbonyl group and the NH_2_ group of the keratin backbone or of an amino acid side group, such as asparagine or lysine. Considering the high number of functional groups in both hydrolyzed keratin and melanin, the formation of a highly cross-linked network is possible. Several studies have shown that the use of glycerol as a softening agent allows keratin to oxidize and is essential in obtaining flexible films [28,41]. Indeed, the ATR spectra in Figure 2 showed increased intensities in the presence of glycerol, specifically of the 1630 cm^−1^ peak, as previously reported [44].

In terms of the mechanical biomimetic properties, the human skin Young’s modulus ranged between 0.42 and 0.75 MPa; KMF40 exhibited 0.5 MPa and therefore complies with real skin modulus. The KMFs’ average toughness is 1.4 Mj/m^3^**,** which is suitable for wearable skin applications such as bio-integrated electronics [6,45]. The ultimate strain of the KMFs ranges between 50 and 80%, which is within the human skin strain of 75% [4]. The addition of melanin results in a higher Young’s modulus and ultimate stress, which supports the assumption that the keratin and melanin are cross-linked. 

When it is applied as a second skin, KMFs are expected to remain intact in an aqueous environment mainly because of the inherent hydrophobicity of the components. On one hand, the addition of melanin caused a decrease in maximal water absorption and higher than expected mass loss. On the other hand, when evaluating the aqueous phase, only low traces of keratin and melanin were found, which supports the suggested assumption of KMF’s cross-linked network. After drying, the film became brittle, which was likely due to glycerol leaching out in the aqueous phase and leaving behind a highly cross-linked matrix between keratin and melanin. 

A major requirement of skin mimicry is the preservation of function, texture, thickness, and pigment. In this work, melanin was added to provide natural skin pigments. UV-VIS measurements exhibited a strong and clear correlation between the melanin concentrations and light transmittance. Human skin naturally blocks 10% of UV light, with the SC being the main barrier [46]. In Table 2, it is shown that KMF0 alone blocked 40% of UV light, while the addition of melanin provided up to 100% UV blockage, which is exceptionally higher than the blockage capacity of natural skin.

Brown to black skin colors and skin tones are directly dictated by melanin concentration and distribution [8,9,10]. As expected, color values intensified as a function of melanin concentration. The CIE L*a*b* values and the chromaticity in Figure 4c showed that the color distribution is almost linear and within ITA° values corresponding to skin tones. Colors can be adjusted by controlling the initial melanin concentration, film thickness, and addition of other materials such as lipids, which are a natural component of the SC. Characterization of these parameters which allow for the modification of aesthetics is crucial for second skin adoption by end-users. 

SC surface appearance is non-homogeneous and varies between individuals, with unique features such as beauty marks, freckles, and pigmentation. While skin tone can be adjusted, skin traits cannot be achieved using a homogeneous film. For this purpose, KMF-based inkjet printing was evaluated for the first time. In other words, a keratin and melanin ink was developed in order to achieve similar properties to KMFs in a flexible form factor allowing for the addition of singular, high-resolution features to otherwise homogenous films. While synthetic melanin has been previously reported as printable [15], to the best of our knowledge, this is the first report of a keratin ink. The keratin solution was successfully formulated into an inkjet ink by controlling the particle size while maintaining its concentration through pH adjustments, without compromising keratin integrity. There were several differences between the KMFs and KMI solutions, such as glycerol concentration and additives, which are not discussed herein. 

The KMI enabled the successful printing of high-resolution images on several substrates, such as Epson paper, PVA paper, and temporary tattoo paper, but it was not yet able to create a detachable film. The ability to print on different substrates could lead to portable skin serving as a printed template on any second skin application, such as in cosmetic or medical usage. The KMI showed uniformity, continuity, and a color gradient that was easily printable using inkjet printing technology with a home printer. The printed objects were of high resolution and possessed corresponding skin colors and skin characteristics. As with the KMFs, color values can be easily calibrated. We aim to be able to print KMFs; however, there are still several restrictions of the ink that need to be further researched in order to truly assess the potential of KMI in the fabrication of second skin. High water content is one of the main issues preventing the printing of KMFs with the ink. There is also currently a focus on developing the ability to print multiple layers while preserving the resolution necessary to obtain a film. Further research into these restrictions is a concrete step toward the future successful development of a second skin that is based on naturally found skin materials and has an emphasis on pigmentation and specific skin traits. 

## 5. Conclusions

In the present work, homogenous keratin and melanin films were prepared as a potential SC-conforming second skin product. Film color was adjustable and mimicked natural human skin tones, provided ample UV blockage, skin-like mechanical properties, and sufficient water stability. The enhanced UV-blocking capacity of KMFs (40%–100%), as compared to natural skin, suggests their potential use in UV-blockage applications. The KMFs were water-based and comprised of non-toxic, cross-linked biomaterials found in human skin. Furthermore, the KMFs can be fabricated using a simple and environmentally friendly method. Future investigation into KMFs should focus on the analysis of skin-function properties, such as water permeation kinetics. Mimicry of skin features should focus on aspects such as texture, pigments, and beauty marks, especially when considering product applications in cosmetology. The presented KMI has good initial properties and some restrictions, which require further research before it can be fully integrated into stand-alone second skin film.

## Figures and Tables

**Table 1:** Concentration of aqueous keratin/melanin after film submersion/removal over one week.

	Keratin [%]	Melanin [%]
KMF0	0	0
KMF500	0	0
KMF250	3.57 ± 3.93	0.06 ± 0.13
KMF100	6.79 ± 0.75	0.16 ± 0.05
KMF40	5.03 ± 3.69	0.87 ± 0.74

**Table 2:** Percent of UV blocked, CIE L*a*b* values, and individual typology angle (ITA)° values of KMFs.

	UV Block [%]	L*	a*	b*	ITA°
KMF0	40.5 ± 0.71	86.34	0.98	−4.51	NA
KMF500	76.5 ± 3.25	82.39	1.74	−0.18	NA
KMF250	81.8 ± 2.43	69.7	3.8	17.23	48
KMF100	91.4 ± 0.93	65.15	5.09	17.14	41
KMF40	97.4 ± 0.46	27.81	17.46	35.15	−32

## Supplementary Materials

The following are available online at https://www.mdpi.com/2073-4360/12/11/2568/s1, Figure S1: SDS-PAGE of the extracted keratins from cashmere wool, Figure S2: KMFs SEM imaging of cross sections, Figure S3: ATR spectra of keratin and melanin, Figure S4: Profiler analysis of one printed layer of KMI.
