# Supplementary material for: Fabrication of Second Skin from Keratin and Melanin
Source: Polymers (Basel). 2020 Nov 2;12(11):2568. doi: 10.3390/polym12112568 (PMC7692603; doi:10.3390/polym12112568)
Supplement: Supplementary file 1 [file polymers-12-02568-s001.pdf]

polymers

Supplementary Materials

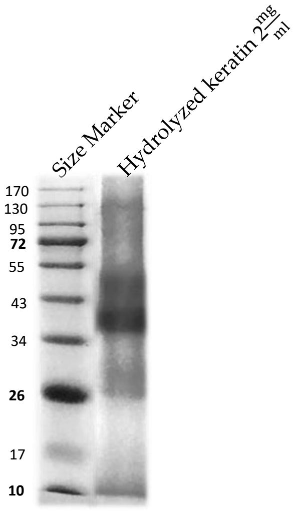

Figure S1. SDS-PAGE of the extracted keratins from cashmere wool. (A) Molecular weight markers; (B) Shindai extract.

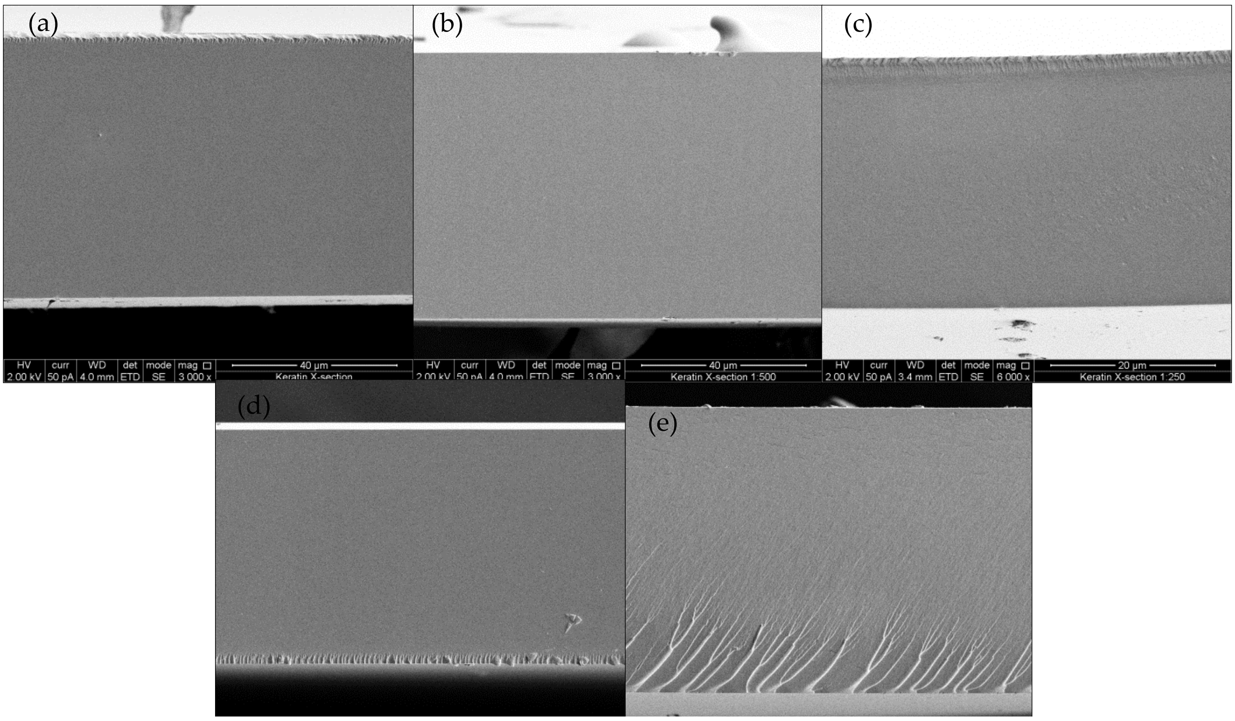

Figure S2. KMFs SEM imaging cross sections (**a-e**): KMF0, KMF500, KMF250, KMF100, KMF40.

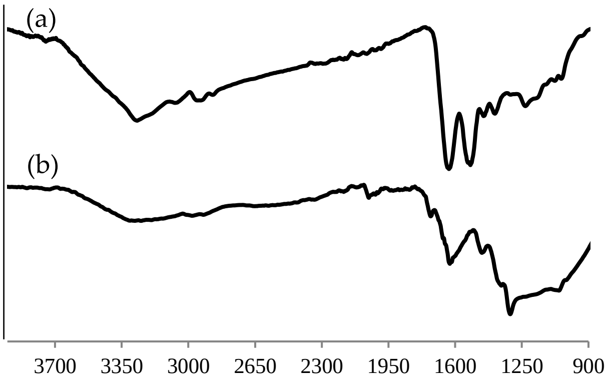

Figure S3. ATR spectra of: (a) keratin, (b) melanin.

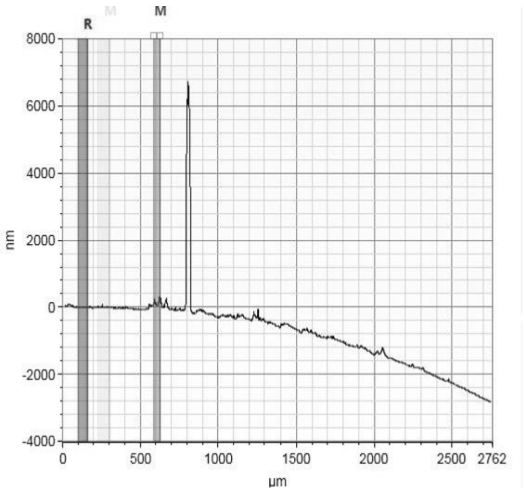

Figure S4: Profiler analysis (Dektak XTTM Stylus Profiler, Bruker, Billerica, MA, USA)) for one printed layer of KMI.
